# Keyphrase Extraction for Technical Language Processing

**DOI:** 10.6028/jres.126.053

**Published:** 2022-03-09

**Authors:** Alden Dima, Aaron Massey

**Affiliations:** 1 National Institute of Standards and Technology, Gaithersburg, MD 20899, USA; 2 University of Maryland Baltimore County, Baltimore, MD 21250, USA

**Keywords:** keyphrase extraction, technical articles, technical language processing

## Abstract

Keyphrase extraction is an important facet of annotation tools that offer the provision of the metadata necessary for technical language processing (TLP). Because TLP imposes additional requirements on typical natural language processing (NLP) methods, we examined TLP keyphrase extraction through the lens of a hypothetical toolkit which consists of a combination of text features and classifers suitable for use in low-resource TLP applications. We compared two approaches for keyphrase extraction: The frst which applied our toolkit-based methods that used only distributional features of words and phrases, and the second was the Maui automatic topic indexer, a well-known academic method. Performance was measured against two collections of technical literature: 1153 articles from Journal of Chemical Thermodynamics (JCT) curated by the National Institute of Standards and Technology Thermodynamics Research Center (TRC) and 244 articles from Task 5 of the Workshop on Semantic Evaluation (SemEval). Both collections have author-provided keyphrases available; the SemEval articles also have reader-provided keyphrases. Our fndings indicate that our toolkit approach was competitive with Maui when author-provided keyphrases were frst removed from the text. For the TRC-JCT articles, the Maui automatic topic indexer reported an F-measure of 29.4 % while our toolkit approach obtained an F-measure of 28.2 %. For the SemEval articles, our toolkit approach using a Naïve Bayes classifer resulted in an F-measure of 20.8 %, which outperformed Maui’s F-measure of 18.8 %.

## 1 Introduction

Technical language processing (TLP) [1, 2] is an adaptation of natural language processing (NLP) to technical domains. It facilitates engineering analyses and research in areas that rely heavily on an abundance of text-based data and metadata. While many NLP-based approaches offer excellent results for tasks such as translation, question answering, unscrambling words, and news article generation [3], they may not be suitable for highly domain-specific uses. Technical text can deviate significantly from the "standard" English used to train and support mainstream NLP methods. These differences have spawned a whole set of domain-specific NLP adaptations that are largely outside of the mainstream applications. For example, to process military aircraft maintenance logs, Bokinsky et al. [4] and McKenzie et al. [5] adapted existing NLP methods to address the challenges of large amounts of inconsistent punctuation, misspellings, and concepts denoted by multiple words by implementing special domain-specific rules.

The classical goals of NLP may also bias it towards resource-intensive approaches while ignoring those more in line with practical technical needs. In NLP, a low-resource setting is one where available resources such as annotated data and algorithms are inadequate for particular analysis tasks [6]. Many technical fields are low-resource settings from the NLP perspective, and the lack of wide-spread vocabulary consensus or adoption is complicated by the existence of facility-specific words [1, 2].

The use of external resources, like Wikipedia, and online tools and dictionaries, may not always be desired, especially in highly competitive industrial environments, where approaches that use such resources could inadvertently reveal sensitive information to competitors. For example, Google collects user search queries and maintains extensive databases on user searches [7]. This information is used to implement its autocomplete feature which suggests searches based on prior related queries [8]. While convenient, autocomplete suggestions are shared among users and can lead to privacy and intellectual property issues [9]. There is even evidence of it playing a role in propagating conspiracy theories related to the coronavirus disease 2019 (COVID-19) pandemic [10]. Nevertheless, Google-style autocomplete is increasingly available in online databases as a research tool [11].

Another example is the accusation by Lina Khan, prior to her current role as chairperson of the U.S. Federal Trade Commission (FTC), that Amazon uses information gleaned from its sellers to compete against them [12]. The online retailer is accused of benefiting from its access to a "vast laboratory" in which it can observe retail trends before exploiting them as a direct competitor. This collection of information is not limited to online marketplaces; the FTC has also found that Internet Service Providers (ISP) routinely collect large amounts of customer information beyond what is needed to provide internet service and use it for their commercial advantage [13, 14]. This information is segmented into focused groups using sensitive information to track customer activities across a wide range of Internet resources and is shared with third parties, often in real time.

Even if online resources are provided using reasonable precautions in an ethical manner, users should hesitate to use them if they are concerned that their online activities could reveal sensitive information. This is highlighted by the current Log4j debacle, which has been called "the most critical vulnerability of the last decade" and "possibly the biggest in the history of modern computing" [15]. Log4j [16] is widely used by online sites to generate system logs, which can contain items of interest to the online site operators, including usage statistics (*i.e.*, who uses the system and how). Attackers can gain full access to a server via the faw, allowing them to steal data and to do much more. The underground economy for stolen data is vast and well organized; the Log4j vulnerability was being exploited by organized crime within in a matter of hours of it being uncovered [17]. State-based actors have also been quick to take advantage of the situation [18].

The variety of ways that information can be collected and used by external actors supports the desire for local, confidential resources in competitive industries. For these reasons, automated, repeatable metadata curation methods such as those based on keyphrase extraction, which promise abundant and consistent metadata, must address additional concerns beyond those addressed by traditional NLP-based systems.

A focus of our efforts is to support annotation tools like Nestor [19], which play an important role in TLP [1, 2]. TLP encourages the use of phrase-based classification of text via tools that rank terms by their estimated importance to a particular corpus, allowing for the rapid annotation of data ranging from maintenance log entries to scientific abstracts. For example, term frequency inverse document frequency (TFIDF) [20, 21] can be used to rank keyphrases for annotating maintenance log entries [22, 23]. Such approaches can operate solely on the available text data and do not rely on outside technical language resources. Their relative ease of implementation and modest resource requirements also facilitates their deployment to industrial analysts, who may not have access to high-end computational facilities.

In addition to appearing in text-based data, technical concepts often have corresponding literature and documentation. These documents can be a fortuitous source of data [24] needed to describe concepts that can be used to annotate concept occurrences found in data sets, including extracted keyphrases. For example, the Vienna Ab initio simulation package (VASP)^1^1Certain commercial equipment or software is identified in this article to foster understanding. Such identification does not imply recommendation or endorsement by the National Institute of Standards and Technology, nor does it imply that the material or software identified is necessarily the best available for the purpose. was described in detail by Kresse and Furthmüller [25]. This article can serve as a source for annotations of VASP-related concepts when they appear in data sets.

Many technical journals also provide keyphrases that describe and organize articles in their collections [26]. These keyphrases are typically noun phrases found in a document that summarize its contents to help potential readers determine the relevance of the document to their needs [26-30]. They can also act as semantic metadata that annotate records, allowing information retrieval systems to index and cluster documents within document collections [27-29, 31-33].

Our goal was to explore the features and classifiers that could be used to build a TLP toolkit that facilitates the creation of classifier-based keyphrase extraction methods. Our approach was based on a set of features, a method for selecting the best features for a given training data set, and a set of classifiers that are trained using the selected features. The classifier that gives the best results for the training data is chosen for the keyphrase extraction method. This toolkit could be realized in a variety of programming languages by TLP tool developers for use in low-resource domains without the need for online resources. We also evaluated combinations of features and classifiers that demonstrate the feasibility of this approach.

Specifically, we (1) developed a toolkit approach for developing keyphrase extraction methods suitable for use in TLP-based annotation tools and to (2) compared them to a well-known method, the Maui automatic topic indexer [34, 35], against two collections of technical documents that also provide keyphrases, which we used as ground truth to evaluate our approach. Unlike methods that make use of document structure [32] or that use online data, our we limited our methods to distributional features of words and phrases within the corpus due to the variety of potential input sources available to TLP annotation tools, as well as to address situations where external resources are not available or appropriate.

We based our work on two collections of technical documents that provide keyphrases: a collection from the National Institute of Standards and Technology (NIST) Thermodynamics Research Center (TRC) and one from the Workshop on Semantic Evaluation (SemEval). While many collections of articles could have been used, the former set was particularly relevant to us because it serves as input data for a long-term TLP-style project at NIST to curate thermophysical data for community use. The TRC is actively working to improve the quality of published thermophysical data in cooperation with five major journals and curates text-based data as part of its efforts [36]. The results of our work have the potential to influence this effort.

The second set of technical documents was taken from Task 5 of the Workshop on Semantic Evaluation 2010 titled "Automatic Keyphrase Extraction from Scientific Articles" [31]. The organizers of this shared task invited developers of keyphrase extraction algorithms to participate and provided them with trial, training, and test data sets.

Using these collections of articles, we evaluated two keyphrase extraction approaches. The first was our proposed approach described in detail in Sec. 3. The second is Maui, a "human-competitive" topic indexer that automatically identifies topics from documents [35].

This paper proceeds as follows. Section 2 surveys related work. Section 3 describes the methodology used in this paper as well as our toolkit approach. Section 4 presents the results, which are discussed in Sec. 5. We explore potential threats to the validity of our work in Sec. 6. Finally, this paper concludes with directions for future research.

## 2 Related Work

Annotation figures prominently in TLP as a means for domain experts to inject their valuable knowledge into the analysis process [1, 2, 22, 37, 38]. During an annotation process, users need keywords, keyphrases, or keyterms that summarize and describe domain concepts. In this paper, we will use the term "keyphrase" to denote all three. These descriptions should be both consistent and meaningful to users so that they yield complete and relevant results.

Another effort at NIST is focused on the generation of a controlled terminology from a collection of technical documents [39] and promises consistent machine-interpretable terms that will be useful in a number of diverse applications. The generated terms differ from the natural language text found in the original documents in that they enforce the use of root terms and structural rules to ensure certain semantics. These terms would be used to annotate items related to a concept and will ensure consistency by eliminating issues with spelling, punctuation, and word order. However, each term will require manual selection from a large vocabulary. Because we wish to automate the generation of metadata from a collection of technical text data, we will instead focus on the automated extraction of keyphrases.

Keyphrases are broadly useful as semantic metadata, as an aid to information retrieval, and for document summarization and clustering [27, 29, 31, 32, 40-43]. Author-provided keyphrases are subjective and require effort on the part of authors who will typically not provide them unless they are required by a publisher [28, 29, 32]. When they do so, authors almost always provide keyphrases as noun phrases [28] that frequently do not appear in their papers [31]. The selection of keyphrases for the existing large majority of documents without them is time-consuming and expensive [26, 28, 29, 33]. For example, Kresse and Furthmüller's article on VASP does not have associated keyphrases and would require a person skilled in their feld to read their paper and select them. As a result, the automatic selection of keyphrases from technical documents is an area of active interest [28, 31].

The keyphrases used during annotation may come from a variety of sources and not exclusively from technical articles: Log files, abstracts, descriptions from web pages, text files, or other short informal texts may be used. As a result, unlike a curated collection of journal articles, there are few guarantees about the structure of the source documents. For this reason, our work used distributional features of words and phrases exclusively because a document describing a technical concept may not have section headers or other structural features that can be exploited by a keyphrase extraction system.

The following are several distributional measures that play an important role in keyphrase extraction and in our work:

## Term frequency (***tf*** ):

the count for each word in a document [20, 21, 44]. Its use in determining keyphrases is based on the notion that frequently used words are more likely to reflect important concepts in a document [20]. The term frequency is often normalized, so that the term frequency for a word *λ_i_* in document *d* is given by: 

$tf(\lambda_{i},d)=\frac{f(\lambda_{i},d)}{\max(f(\lambda_{1},d),...,f(\lambda_{n},d))}$ (1) 

where *f* (*λ_i_, d*) is the number of occurrences of word *λ_i_* in document *d*.

**Document Frequency **(*df* ):

a count of the documents in a text collection in which a word appears [21]. The document frequency can be normalized and expressed as:

$df(\lambda )=\frac{n_{\lambda }}{N_{D}}$(2)

where *n_λ_* is the number of files containing word *λ* and *N_D_* is the total number of documents. Document frequency is used as a measure of the informativeness of a word; a word that appears in many documents offers less ability to discriminate between documents than a word that appears in few documents [21].

**Inverse Document Frequency** (*idf* ):

the logarithm of the reciprocal of the normalized document frequency:

$idf(\lambda )={log}_{2}\left[\frac{1}{df(\lambda )}\right]$(3)

Like the document frequency, the inverse document frequency is used as a measure of the informativeness of a word and can be used to penalize words that appear in many documents.

**Term Frequency Inverse Document Frequency** (*tfidf* ):

the product of the normalized frequency of a word in a document *d* to the word's inverse document frequency [20, 21]:

*tfidf* (*λ,d*) = *tf* (*λ,d*) * ·idf* (*λ*) (4)

The use of the inverse document frequency penalizes words that appear in many documents to address the concern with term frequency where words appear frequently across large portions of the documents in a collection lack discriminative power [44].

**Lift:** a data mining correlation measure; it is the fraction of the results containing all members of a desired set to the product of the fractions containing individual members [45]. When applied to words in a text collection, it can be expressed as

$lift(\lambda_{1},...,\lambda_{n})=\frac{n_{\lambda_{1}},...,\lambda_{n}}{\Pi_{i=1}^{n}n_{\lambda_{i}}}$(5)

where $n_{\lambda_{1}},...{,}_{\lambda n}$ is the count of documents containing all of the words *λ*_1_, *. . ., λ_n_*, and *n_λ__i_* is the count of the documents containing word *λ_i_*. We use variants of lift as measures of phraseness, which is the lexical cohesion of the words in a phrase [32]. They give measures of the likelihood of words to appear together and imply a semantic relationship.

Because they play an important role in what follows, we also briefly describe three information-retrieval measures [44, 46] that are used to describe the performance of keyphrase extraction methods:

**Precision:**

the fraction, *p*, of the total items retrieved that are relevant:

$p=\frac{TP}{TP+FP}$(6)

where *TP* is the number of the true positives, and *FP* is the number of false positives. For our work, *TP* represents the number of noun phrases that are identified as keyphrases by a keyphrase extraction method that also appear in the human-generated ground truth, and *FP* represents the number of noun phrases that are identified as keyphrases that do not appear in the ground truth data set.

**Recall:**

the fraction, *r*, of the relevant items that are retrieved:

$r=\frac{TP}{TP+FN}$ (7)

where *TP* is defined above, and *FN* represents the number of keyphrases in the ground truth that are not identified as such by the keyphrase extraction method.

*F* **-measure:**

the harmonic mean of the precision and recall, which is defined as:

$F=\frac{2}{\frac{1}{p}+\frac{1}{r}}$(8)

The *F*-measure combines the precision and recall into a single measure for which the value is closer to the smaller of the two.

As can be seen by their definitions, these measures depend on the ground truth. For keyphrase extraction, this human-generated ground truth is subjective. It can be simultaneously incomplete and also contain keyphrases that the extractive methods evaluated in this work can never produce. Evaluations relying on this type of reference data set can only provide indications rather than absolute measures of performance. These issues are discussed in Sec. 5 and Sec. 6.

Kea [33] was one of the earliest supervised keyphrase extraction systems and serves today as a basis for newer methods such as Maui [35]. It calculates two features for each candidate keyphrase in a training set: term frequency inverse document frequency (*tfidf*) and the location of the first appearance of the candidate in the text [33]. It then trains a Naïve Bayes classifier for use with new documents. Classifiers have continued to play a role in supervised keyphrase extraction systems [32, 35, 47].

Subsequent work has focused on an increased variety of features and machine learning techniques. Hulth [42] examined the effects of stemming, three term-selection approaches (*n*-gram, chunking, and patterns), and four features: term frequency, document frequency, location of first appearance, and sequence of part-of-speech (POS) tags. She found that the best *F*-measure was obtained using *n*-grams with POS tags, the best precision was obtained using chunking with POS tags, and the best recall was achieved using POS patterns. She also found that stemming in each case led to the best results.

In 2010, the International Workshop for Semantic Evaluation's Task 5, "Automatic Keyphrase Extraction from Scientific Articles" [31] was an assessment of the state-of-the-art for scientific keyphrase extraction. Participants were asked to develop methods for extracting keyphrases from a collection of 244 articles taken from the Association for Computing Machinery (ACM) Digital Library falling under four classifications codes:

1. Distributed Systems,

2. Information Search and Retrieval,

3. Distributed Artificial Intelligence - Multiagent Systems, and

4. Social and Behavioral Sciences - Economics.

Custom unsupervised and supervised methods were used to select keyphrases from *n*-grams (*n * ≤3) generated from the articles using *tfidf* as a feature. The unsupervised methods ranked candidates by their *tfidf* scores, while the supervised ones used author- and reader-selected keyphrases as ground truth and trained Naïve Bayes and maximum entropy classifiers to select keyphrases. The task organizers reported an *F*-measure of 15.1% for the unsupervised custom method and 14.7% for both supervised methods using test data consisting of the top 15 combined author- and reader-selected keyphrases [31].

The best performing keyphrase extraction method in SemEval Task 5 was HUMB [32] which had an *F*-measure of 27.5% for the top 15 combined test data [31]. HUMB uses three sets of features to identify keyphrases [32]:

1. Boolean structural features consisting that describe the presence of a candidate term in the title, abstract, introduction, section titles, conclusion, and references;

2. distributional features for a candidate term relative to the document and collection; and

3. lexical and semantic features, including the length of a candidate term and the probability that a candidate term appears as an anchor in Wikipedia.

The distributional features used by HUMB were the most similar to the features used in this work. The generalized Dice coefficient was used there to measure the phraseness of a candidate term, which depends on the frequencies of the term and its individual words as well as the number of words in the term [32]. The informativeness of a term was determined using *tfidf*, and the frequency of the term in the collection was used to quantify its role as a keyphrase [32]. The Dice coefficient is a set similarity measure that originated from ecology and serves to quantify the similarity of two samples [48]. It serves a different purpose from lift, which is used to identify set elements that co-occur.

We were not able to obtain HUMB for this work. We instead used the Maui automatic topic indexer, which also participated in SemEval Task 5 and is available as open-source software [31, 34, 35]. It ranked ninth overall among the participants in SemEval Task 5 with an *F*-measure of 20.6% for the top 15 combined author- and reader-selected keyphrases [31].

Maui is derived from Kea and Waikato Environment for Knowledge Analysis (WEKA) [35]. It is based on *n*-gram term selection and filters extracted *n*-grams based on the presence of an external vocabulary [35]. If no vocabulary is used, then Maui filters *n*-grams based on the location of stop words found in the *n*-gram. With an external vocabulary such a thesaurus or Wikipedia, Maui uses either search or statistics to filter the candidates [35]. Compared to Kea, Maui calculates a large number of features for each candidate keyphrase. In addition to *tfidf* and the first appearance of a term, Maui also computes:

1. training data term frequency,
2. keyphrase length,
3. node degree and semantic relatedness,
4. likelihood of being a Wikipedia link,
5. spread of locations in a document, and
6. inverse Wikipedia linkage.

## 3 Methodology

This section serves two purposes. The first is to detail our methodology. The second is to provide a glimpse at the features and functionalities on which our hypothetical toolkit depends. These will have to either be made available to a realization of our toolkit or implemented by the toolkit.

As mentioned in Sec. 1, we used two collections of articles for this work. They are summarized as follows:

1. TRC-JCT: A collection of 1153 curated articles in portable document format (PDF) from the Journal of Chemical Thermodynamics (JCT) [49] along with ThermoML [50] files containing keyphrases from the ThermoML Archive [51]; and
2. SemEval: A collection of 244 articles curated from the Association for Computing Machinery's (ACM) Digital Library available from the SemEval-2 website [52] and from the Maui developer's GitHub repository [53].

**Fig. 1:**
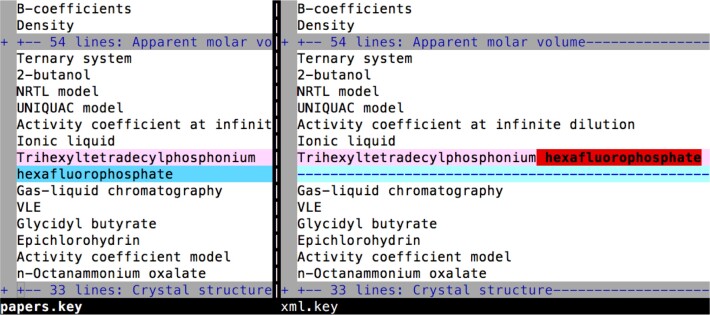
An example of a modification made to a TRC-JCT keyphrase during the TRC curation process.

Using these two collections of technical articles, we evaluated two keyphrase extraction approaches:

Toolkit: Our proposed approach, which uses features computed from the extracted text to train WEKA classifiers [54]; and

Maui: The Maui automatic topic indexer, standalone version 1.1 [34, 35, 55].

Our toolkit approach requires NLP to parse, tag, extract, and lemmatize noun phrases. We created data sets consisting of the lemmatized noun phrases along with their statistical features. We then used WEKA to select attributes and train classifiers to identify the keyphrases. Maui is a "human-competitive" topic indexer that automatically identifies topics from documents [35]. Maui directly processes the input text and does not need separate preprocessing, data set generation, and attribute selection steps.

We began our work with the TRC-JCT collection and developed our toolkit approach in an exploratory fashion. We then obtained the SemEval collection and used both our toolkit approach and Maui to confirm our results. We will describe these steps in more detail as follows. Section 3.1 will discuss the preparation and structure of the TRC-JCT data set. Section 3.2 will discuss the evaluation of the TRC-JCT data set with both our toolkit approach and with Maui. Section 3.3 will do the same for the SemEval data set.

## 3.1 Preparation and Structure of the TRC-JCT Data Set

We obtained the TRC-JCT journal articles as PDF documents via the NIST TRC in Boulder, CO [56]. These articles are copyrighted material and are available to us via an institutional subscription. Their corresponding ThermoML fles are produced by the TRC under an arrangement with the publisher and are available from the TRC ThermoML Data Archive website^2^2https://trc.nist.gov/ThermoML.html. We provide instructions for re-assembling our data set in the Appendices (Secs. 8 and 9).

We then used the Linux pdftotext [57] utility to extract the text from the PDF documents in American Standard Code for Information Interchange (ASCII) format. We also downloaded their corresponding ThermoML files from the ThermoML Data Archive [51].

The PDF file names did not match the ThermoML file names because the former were based on the article title, and the latter were derived from the article digital object identifier (DOI). We used a Perl script to examine the extracted text, identify the appropriate DOIs, and rename the files.

Figure captions interrupted the regular text in the extracted text files. We used a second Perl script to identify the figure captions and move them to the bottom of the extracted text so that the text could be parsed correctly.

Some keyphrase extraction methods, such as Kea and Maui, use the location of the first occurrence of a candidate phrase to detect keyphrases [33, 35]. Our toolkit approach does not. The use of the location of the first occurrence of a keyphrase assumes certain things about document structure that may not generally hold in TLP, especially for shorter documents. We believe that the presence of author-provided keyphrases at the beginning of papers leads to results that are not representative of the underlying method's performance in these more general settings. We instead focused on statistical features to identify keyphrases.

Because the TRC-JCT articles embed the author-provided keyphrases near the beginning of the article, we wanted to understand the effect of these embedded keyphrases on the two approaches. We used a variant of the Perl script that moved figure captions to remove the author-provided keyphrases found at the beginning of each article to produce a second training set without embedded keyphrases.

For the toolkit approach, several preprocessing steps were then necessary to generate the data needed to train and test the classifiers. We used the Python spacy.io NLP package [58] to parse the text and extract lemmatized noun phrases. We then selected adjectives and nouns from the phrases under the assumption that most relevant noun phrases would consist of adjectives and nouns.

Both the journal website [49] and conversations with TRC staff members suggested that the article keyphrases are provided by individual authors during manuscript submission. These keyphrases appear at the beginning of the journal articles. We also obtained TRC-created ThermoML files corresponding to each journal article. The ThermoML files contain slightly edited versions of the author-provided keyphrases. The most significant change was to ensure that there were no embedded newlines in the keyphrases that would cause us to interpret one keyphrase as two independent keyphrases (see Fig. 1). There were some small variations in punctuation and occasional modifications of some phrases to suit the needs of the TRC curation process. We chose to use the TRC-curated keyphrases because the changes made simplified the process of generating the TRC-JCT data set. We extracted the keyphrases from the extensible markup language (XML)-based ThermoML files. These keyphrases are located in <sKeyword> elements [59]. We extracted them using a simple IPython notebook [60] using regular expressions. We lemmatized the keyphrase and kept the words tagged as nouns and adjectives. We then compared the resulting normalized keyphrases to each noun phrase while generating the data set to identify matches.

We generated the data set used to train the classifiers using an IPython notebook and did some additional post-processing with an R script [61]. The R script also allowed us to generate separate training and test data sets from the same underlying data set.

The columns of the TRC-JCT training data contained:

1. the document file name,
2. the noun phrase,
3. statistics for the noun phrase, and
4. an identifier denoting whether the noun phrase was a keyphrase.

We collected 10 different statistics for each noun phrase. Four of the statistics are well known in the NLP and data-mining communities. We augmented them with six variants. Some altered an NLP statistic to account for individual occurrences of the keyphrase's constituent words in the document. Three of the variants were derivatives of the lift measure used in association rule mining. These statistics are defined below. Those that are denoted with a Φ subscript are statistics for the noun phrase (denoted by *ϕ*) as it appears in the text, while those with Λ subscripts are variants that take into account the appearance of the phrase's individual words (denoted by *λ*) outside of the phrase. Example values for these statistics are shown in Table 1.

**Term frequency (** *tf*_Φ_):

a normalized count of each candidate noun phrase ϕ in a document *d*:

*tf*_Φ_$(\phi_{i},d)=\left[\frac{f(\phi_{i},d)}{\max(f(\phi_{1},d),...,f(\phi_{n},d)}\right]$(9)

**Log Lift (** *llift* **):**

a variation of the lift measure described in Sec. 2, defined as the logarithm of the ratio of the fraction of the noun phrases containing a candidate phrase to the product of the fractions of the noun phrases containing an individual word of the candidate phrase.

*llift*_Λ_$(\phi )={log}_{2}\left[\frac{n_{\phi }}{\Pi_{i=1}^{n}n_{\lambda_{i}}}\right]$(10)

where $n_{\phi }$, is the count for the candidate phrase ϕ and *n_λ_* is the number of phrases containing an individual word from the candidate phrase. This and two other statistics below (*ldlift*_Φ_ and *ldlift*_Λ_) are base 2 logarithms of other statistics much in the same vein as *logDice* which is another *log*_2_ statistic wherein the use of the logarithm helps to ensure that the values are stable and scale well across corpus sizes [62].

**Document Frequency (***df*_Λ_, *df*_Φ_**):**

defined using two variants, one word oriented and the other phrase oriented:

1. a normalized count of the documents in which all the individual words *λ*_1_, *. . . λ_n_* of a noun phrase appear: 

*df*_Λ_$(\lambda )=\frac{n_{\lambda_{1},...,\lambda_{n}}}{N_{D}}$(11)

where $n_{\lambda_{1},...,\lambda_{n}}$ is the number of documents containing the phrase's words and *N_D_* is the total number of documents; and

2. a normalized count of the documents in which the noun phrase appears [20, 21]:

*df*_Φ_$(\phi )=\frac{n_{\phi }}{N_{D}}$(12)

where $n_{\phi }$ is the number of documents containing the phrase ϕ, and *N_D_* is the total number of documents.

**Inverse Document Frequency (***idf*_Λ_, *idf*_Φ_**):**

defined using two variants corresponding to the two definitions for document frequency above:

1.The logarithm of the reciprocal of the document frequency:

*idf*_Λ_$(\phi )={log}_{2}\left[\frac{1}{df_{\Lambda }(\lambda_{1},...,\lambda_{n}}\right]$ (13)

where *ϕ* is a noun phrase consisting of words *λ*_1_, *. . ., λ_n_*, and *df* is the normalized count of documents in which the words appear, though not necessarily together as the noun phrase.

2. the logarithm of the reciprocal of the second variant of the document frequency:

*idf*_Φ_$(\phi )={log}_{2}\left[\frac{1}{df_{\Phi }(\phi )}\right]$ (14)

where *ϕ* is a noun phrase and *df* is the normalized count of documents in which the phrase appears.

**Log Document Lift (***ldlift*_Φ_, *ldlift*_Λ_**):**

defined using two document-oriented correlation measures inspired by the lift metric:

1. the logarithm of the ratio of the fraction of documents containing the noun phrase to the product of the fractions containing an individual word from the noun phrase:

*ldlift*_Φ_$(\phi )={log}_{2}\left[\frac{df_{\Phi }(\phi )}{\Pi_{i=1}^{n}df_{\Lambda }(\lambda_{i})}\right]$(15)

where ϕ is a phrase consisting of words *λ*_1_, *. . ., λ_n_*; and

2. the logarithm of the ratio of the fraction of documents containing all of the individual words of a noun phrase to the product of the fractions of documents containing an individual word of the noun phrase:

*ldlift*_Λ_$(\phi )={log}_{2}\left[\frac{df_{\Lambda }(\lambda_{1},...,\lambda_{n})}{\Pi_{i=1}^{n}df_{\Lambda }(\lambda_{i})}\right]$ (16)

where *ϕ* is a phrase consisting of words *λ*_1_, *. . ., λ_n_*.

**Term Frequency Inverse Document Frequency (***tfidf*_Λ_, *tfidf*_Φ_**):**

defined using two versions of the *tfidf* measure:

1. the product of the normalized frequency of a noun phrase ϕ to its inverse document frequency [20, 21]:

*tfidf*_Λ_$\left(\phi ,d)=tf_{\Phi }(\phi )\cdot idf_{\Lambda }(\phi ,d\right)$ (17)

and

2. the product of the normalized frequency of a noun phrase *ϕ* to the second variant of inverse document frequency:

*tfidf*_Φ_$\left(\phi ,d)=tf_{\Phi }(\phi )\cdot idf_{\Phi }(\phi ,d\right)$ (18)

As this was an exploratory study, we do not claim that these statistics are the best choices for our toolkit but instead that they served as a reasonable starting point for our investigation. In selecting them, our intention was to have multiple statistics available for the occurrence and co-occurrence of candidate phrases and their constituent words. It should also be noted that we applied feature subset selection, as described below in Section 3.2, and that our classifiers only used the subset of these collected features chosen by our approach.

Table 1 gives some example values for the 10 statistics described above for three arbitrary noun phrases taken from different documents to give the reader a sense of typical data; they are for illustrative purposes only. The first row of Table 1 contains the statistics for the phrase "cyclohexane." Note that its log lift (*llift*_Λ_) value is zero because it is a single-word phrase. The first document frequency value is greater than the second document frequency value because the word "cyclohexane" appears in other noun phrases such as "cyclohexane mixture" and "benzene cyclohexane." The second document frequency value only accounts for noun phrases that match "cyclohexane."

**Table 1:** Illustrative example statistics for selected noun phrases.

Data Set	Counts Noun Phrases Keyphrases		*tf*_Φ_		*llift*_Λ_		*df*_Λ_		*df*_Φ_		*idf*_Λ_		*idf*_Φ_		*ldlift*_Φ_		*ldlift*_Λ_		*tfidf*_Λ_		*tfidf*_Φ_	
2012.07.018 2014.07.005 2013.09.018		cyclo- hexane polymer solution ion solvent interaction		0.015 0.010 0.006		0.000 1.376 3.071		0.082 0.097 0.281		0.069 0.005 0.023		3.617 3.364 1.831		3.867 7.586 5.416		0.000 0.214 0.525		-0.251 -4.009 -3.060		0.053 0.032 0.011		0.056 0.072 0.032	

**Table 2:** Noun phrase and keyphrase counts for TRC-JCT data sets.

Data Set	Counts Noun Phrases Keyphrases	
Overall	449,981	4162
Training with keyphrases	315,240	2999
Training without keyphrases	314,493	2970
Test	134,741	1163

### 3.2 Evaluation of the TRC-JCT Data Set

We evaluated our toolkit approach with the TRC-JCT data using WEKA. First, we selected attributes with WEKA's Correlation-based Feature Subset Selection method (CfsSubsetEval) [46]. We evaluated four WEKA classifiers using test set-based validation:

1. Naïve Bayes: assumes that predictors have independent distributions [63];
2. J48: a decision tree classifier based on the C4.5 classifier [46, 63, 64];
3. random forest: builds an ensemble of decision trees that use different subsets of the predictors [65]; and
4. AdaBoost M1/J48: uses a multistage process to train successive J48 classifiers so that currently misclassified data is weighted to increase its importance in future stages [65, 66].

We used the WEKA-provided default parameters for each classifier. We will refer to the four methods of our approach as follows:

1. Toolkit(NB): toolkit approach using Naïve Bayes classifier;
2. Toolkit(J8): toolkit approach using J48 decision tree classifier;
3. Toolkit(RF): toolkit approach using random forest classifier; and
4. Toolkit(AB): toolkit approach using AdaBoost metaclassifier with J48 decision tree classifier.

**Table 3:** Test Set Evaluation of TRC-JCT training data.

Method	Embedded Keyphrases	Method Performance Precision, % Recall, % *F*-measure, %		
Toolkit(NB)	Yes	15.8	40.9	22.8
Toolkit(J48)	Yes	64.0	16.3	26.0
Toolkit(RF)	Yes	47.0	20.6	28.7
Toolkit(AB)	Yes	37.6	22.4	28.1
Maui	Yes	31.2 *±* 15.2*^a^*	48.0 *±* 22.6*^a^*	37.8
Toolkit(NB)	No	16.0	41.5	23.1
Toolkit(J48)	No	61.9	16.6	26.2
Toolkit(RF)	No	48.0	19.3	27.5
Toolkit(AB)	No	40.5	21.7	28.2
Maui	No	24.2 *±* 13.9*^a^*	37.3 *±* 20.8*^a^*	29.4

a Maui computed uncertainty: ± two standard deviations

### 3.3 Preparation and Evaluation of the SemEval Data Set

The SemEval data set required different handling for the toolkit approach because the text and keyphrases were provided in ASCII format. We used the same process as the TRC-JCT data to extract the noun phrases and generate the training set. We chose to use the combined author- and reader-selected keyphrases from the Maui-provided SemEval data set as the test data (see below). We structured the SemEval data set to mirror the structure of the TRC-JCT training and test data. The data set was divided by its developers into a training set of 144 articles and a test set of 100 articles [31].

We chose not to use the evaluation script provided with the SemEval data set because it assumes a regression-based method that provides a ranked list of keyphrases. Instead, we evaluated the toolkit approach with the SemEval data set following the same procedure as the TRC-JCT data set (see Sec. 3.2).

We obtained the SemEval data set structured in a manner suitable for use by the Maui automatic topic indexer from one of the Maui developer's GitHub repositories [53]. This version of the SemEval data set provided a combined author- and reader-selected keyphrase test set, which was used in our evaluation of Maui. We were able to evaluate Maui against the TRC-JCT training and test sets with no modifications. Maui does not require preprocessing because most of this functionality is built into the indexer. It also has a test mode, which will evaluate its results against a test set and report the relevant statistics.

## 4 Results

In this section, we present the results from our evaluations of our toolkit approach and Maui using the data sets generated from the two collections of technical journal articles. Section 4.1 presents the results for both approaches with TRC-JCT data set and Sec. 4.2 presents those for the SemEval data set.

### 4.1 TRC-JCT Data Set

We created our two training sets from the 828 TRC-curated JCT articles published through 2014. The test data set contained 325 TRC-curated JCT articles from 2015 and 2016. Table 2 gives noun phrases and keyphrase counts for the TRC-JCT data sets.

We selected the following four attributes using the WEKA CfsSubsetEval attribute selection method:

**Table 4:** Test set evaluation for SemEval training data.

Method	Method Performance Precision, % Recall, % *F*-measure, %		
Toolkit(NB)	17.8	24.8	20.8
Toolkit(J48)	0.0	0.0	0.0
Toolkit(RF)	28.6	1.3	2.5
Toolkit(AB)	9.9	2.6	4.1
Maui	27.0 *±* 16.3*^b^*	14.5 *±* 8.8*^b^*	18.8

b Maui computed uncertainty: ± two standard deviations

*tf*_Φ_, *ldlift*_Λ_, *tfidf*_Λ_, and *tfidf*_Φ_ (see Sec. 3.2). The first four rows of Table 3 give the results for the four methods of our toolkit approach with the TRC-JCT data set containing embedded keyphrases. The results for Maui with the same data set are given in the fifth row. Maui reported an *F*-measure of 37.78%. For the toolkit approach, we obtained an *F*-measure of 28.7% with Toolkit(RF). We obtained the highest precision with Toolkit(J48) (64%) but this corresponded to the lowest recall (16.3%) for the toolkit approach with this data set and yielded an overall *F*-measure of 26%. Toolkit(NB) yielded the highest recall (40.9%) but the lowest *F*-measure (22.8%).

The bottom half of Table 3 shows our results for the TRC-JCT training data without embedded keyphrases. The results show that when the embedded keyphrases at the beginning of the paper are removed, the performance of Maui drops significantly because it uses the location of the first appearance of a keyphrase as a feature and author-provided keyphrases are typically placed at the beginning of a paper. The first through fourth rows give our results for the four methods in our toolkit approach when author-provided keyphrases are left embedded in the text. These results are essentially the same as those in the corresponding sixth through ninth rows of Table 3, where the same author-provided keyphrases were removed. The fifth and last rows give Maui's performance under the same conditions; its performance decreased significantly when the author-provided keywords were removed (the *F*-measure dropped from 37.8% to 29.37%). For the toolkit approach, we obtained the best overall results with Toolkit(AB), which yielded an F-score of 28.2%. As with the TRC-JCT data set with embedded keyphrases, Toolkit(J48) gave the highest precision (61.9%) and Toolkit(NB) had the highest recall (41.5%).

### 4.2 SemEval Data Set

The SemEval training set consisted of 83,815 non-keyphrase and 1378 keyphrase noun phrases. The test set consisted of 58,899 non-keyphrase noun phrases and 922 keyphrases. We selected the following three attributes using the WEKA CfsSubsetEval attribute selection method: *tf*_Φ_, *tfidf*_Λ_, and *tfidf*_Φ_ (see Sec. 3.2). The first four rows of Table 4 give the results for the four methods of the toolkit approach, and the fifth row has Maui's results. Toolkit(NB) had an *F*-measure of 20.8% with a recall of 24.8%. Maui reported an *F*-measure of 18.84%. Toolkit(RF) yielded a higher precision (28.6%), but the *F*-measure was significantly lower (2.5%). Toolkit(J48) did not produce useful results (*F*-measure = 0.0).

## 5 Discussion

A key motivation of this work was to determine an effective keyphrase extraction implementation approach for TLP annotation tools such as Nestor. While Nestor started in the manufacturing maintenance domain where the documents are usually very short, its use is being expanded to other domains involving longer and more complex text, such as paragraphs and sections, where it will benefit from more sophisticated keyphrase extraction methods. The academic methods presented in the literature are often unavailable or not maintained in the future. The availability of high-quality, open-source NLP and machine learning libraries encourages us to consider whether we can effectively implement keyphrase extraction systems that ft well into our existing architectures.

One immediately striking aspect of keyphrase extraction is the low scores associated with state-of-the-art algorithms which have maximum *F*-measures less than 30%. This stands in contrast with the expectations for most other machine-learning-based tasks, where *F*-measures can exceed 90% for classification tasks and results from the fundamental nature of ground truth as it relates to keyphrase extraction. As we will discuss in Sec. 6, our ground truth consisted of human-provided keyphrases and is inherently subjective, as reflected in the differing sets of SemEval ground truth keyphrases that were provided from two sources (authors and readers). Keyphrases identified by an extraction method that do not appear in the ground truth will increase the *FP* term in the denominator of Eq. (6) and consequently reduce the value of the *F*-measure given by Eq. (8).

Keyphrase extraction algorithms are also constrained by the fact that they can only identify keyphrases that exist within a document. Unlike humans, they have no ability to generate novel yet relevant keyphrases. Yet, despite instructions to only use keyphrases existing within a document, a significant number of the ground truth keyphrases were novel (see Sec. 6) and are therefore out of reach of keyphrase extraction algorithms. The presence of these novel keyphrases in the ground truth can only serve to lower the scores, because they will increase the *FN* term in the denominator of Eq. (7) and also reduce the value of the *F*-measure given by Eq. (8).

In the remainder of this section, we will discuss effects due to the presence of author-provided keyphrases (Sec. 5.1), corpus size (Sec. 5.2), and the choice of classifier used (Sec. 5.3).

### 5.1 Presence of Author-Provided Keyphrases

The practical use of a keyphrase extraction method is affected by the presence of author-provided keyphrases in the beginning of technical documents because users will not remove embedded keyphrases prior to applying a method. Methods such as Maui use the location of candidate terms as a feature [35]. We did not use the first occurrence of a candidate phrase as a feature to our approach because we expected that TLP applications will use shorter, more varied text where such assumptions about the structure of the document do not hold. We also believe that the presence of user-provided keyphrases in evaluation data sets can lead to results that are not representative of the underlying method's performance in more general settings. Though the organizers of SemEval 2010 Task 5 did not include the author-provided keyphrases in their extracted text [31], we created two versions of the TRC-JCT training data, both with and without the embedded author-provided keyphrases. The results in Table 3 show that Maui's performance was significantly better than our Toolkit(AB) approach when the author-provided keyphrases were left embedded at the beginning of the articles. The results shown in the bottom half of Table 3 and in Table 4 reveal that our best performing toolkit approaches, Toolkit(AB) and Toolkit(NB), were competitive with Maui when the embedded keyphrases were not present in the text. While the performance of our toolkit approaches did not change significantly, those of Maui did when these author-provided keyphrases were removed from the beginning of the papers. The presence of these keyphrases biases the algorithms that use the first keyphrase location heuristic to favor these keyphrases. In TLP, the focus is on shorter texts, especially for annotation tasks, and the frst location of a keyphrase is not as useful. We were able to achieve comparable performance with our simpler toolkit methods, which are within ready reach of TLP developers. These methods can be readily encoded into their existing TLP workflows and do not require integration of different implementation technologies (for example, interfacing Python TLP workflows with Maui's Java-based implementation).

### 5.2 Corpus Size

The size of the corpus affected the overall performance of the keyphrase extraction methods. Precision increased slightly with the decreased article collection size, while recall decreased more significantly. The larger of our two collections, TRC-JCT, contained 1153 articles, while the other, SemEval, had 244 articles (Sec. 1). Maui had the best average *F*-measure (24.1%) across the two data sets (bottom half of Table 3 and Table 4) and Toolkit(NB) had the second best (21.8%). We do not consider the remaining toolkit approaches here because they performed poorly with the SemEval data set; their *F*-measures were below 5%. The average precisions for Maui and Toolkit(NB) increased from 24.2% to 27.0% and from 16.0% to 17.8%, respectively, between the larger TRC-JCT data set and the smaller SemEval data set. In contrast, their average recalls dropped from 37.3% to 14.5% and from 41.5% to 24.8%, respectively. Higher recall scores are beneficial for our application because the larger pool of extracted keyphrases will offer more useful keyphrases to the users. This suggests that we will have to curate a large collection of literature and documentation to support effective use of keyphrase extraction for annotation.

### 5.3 Choice of Classifier

The Naïve Bayes classifier used in Toolkit(NB) performed well across both document collections, giving an average *F*-measure of 21.8% across the results shown in the bottom half of Table 3 and Table 4. While Toolkit(J48), Toolkit(RF), and Toolkit(AB) out-performed Toolkit(NB) with TRC-JCT data, they performed poorly with the SemEval data (Table 4), resulting in average F-scores across both data sets of 13.1%, 15.0%, and 16.2%, respectively. Recall is important because it determines the number of candidate keyphrases presented to the user; Toolkit(NB) consistently had the highest recall scores among the toolkit approach methods (see Tables 3 and 4). Because of its stable performance and recall scores, we will favor the use of the Naïve Bayes classifier for annotation-related keyphrase extraction. From a research standpoint, there is more work to be done in terms of classifier selection. The Naïve Bayes classifier is suitable for this task because its consistent performance across both document collections suggests that it will provide annotators with a uniform keyphrase suggestion experience, which increases their confidence in its results versus classifiers that show greater variation. Its higher recall scores relative to the other classifiers used in our toolkit approach means that the Naïve Bayes classifier will identify more useful candidate keyphrases for annotators.

## 6 Threats to Validity

We now consider three potential threats to the validity of our work. Construct validity is concerned with whether the measurements in an empirical investigation correctly assess the underlying phenomena that are the subject of study [67, 68]. It is of central importance in psychology where the variable to be measured is often not directly observable [69]. Construct validity is an area of concern for our work. We evaluated the performance of two keyphrase extraction approaches using human-provided keyphrases as ground truth.

The manual assignment of keyphrases is a subjective task. The organizers of the SemEval keyphrase extraction task used both author- and reader-provided keyphrases as ground truth; these lists of keyphrases differed [31]. Furthermore, the organizers discovered that 19% of the author-provided keyphrases and 15% of the reader-provided keyphrases did not appear in the articles despite specific instructions to the readers to only choose existing phrases [31]. Evaluation using human-generated ground truth does not address the question of whether the keyphrases selected are useful. User studies that focus on an area of application for keyphrases provide a better means of evaluating the performance of keyphrase extraction methods for that application.

Internal validity is concerned with the effects of confounding variables on conclusions drawn by a study [67, 68]. Though the establishment of causal relationships was not our goal, with such a small data set, we have concerns about the internal validity of our work. In Sec. 5.2 above, we claim that the size of the collection affects the results: The recall will increase faster with collection size than the precision will decrease leading to an overall improvement in performance as gauged by the *F*-measure. There were, however, effects that we are not able to characterize with our work. The TRC-JCT collection is focused on a narrow domain (chemical thermodynamics) and is published in a single journal while the SemEval collection contains a mix of four sub-domains within computer science taken from the ACM Digital Library. The SemEval articles are likely from different journals. In the future, we plan to examine the effect of collection size by using subsets of differing sizes from several large collections. This approach would allow us to be more certain of the causal relationship between collection size and keyphrase extraction performance for the two approaches that we investigated.

External validity is concerned with whether the study results are generalizable throughout a domain [68]. We used two document collections of differing sizes taken from two different technical domains. We also evaluated two approaches to extract keyphrases. Nonetheless, the generalizability of our results may be called into question. Our work suggests that our toolkit approach to keyphrase extraction is competitive with a well-known academic approach despite resembling some of the older classifier-based approaches. With only two data collections for analysis, it is difficult for us to say much about the generalizability of our approach, but we plan to examine additional collections of technical documents to determine whether our approach continues to perform well. We only evaluated Maui in its stand-alone mode. Maui can also reference external resources, such as Wikipedia, to evaluate candidate keyphrases [35]. We argued in the introduction that the use of external resources may not be desirable in certain situations but we can examine this approach in the future for less sensitive domains as the TLP community develops shared domain-specific vocabulary resources.

## 7 Summary and Future Work

Motivated by data annotation for TLP applications, we explored an approach for technical keyphrase extraction based on a hypothetical toolkit that could be used by developers within their current applications. We compared our approach for keyphrase extraction with another approach from academia (Maui) against two collections of technical literature. Both the TRC-JCT and SemEval collections have author-provided keyphrases available; the SemEval articles also have reader-provided keyphrases. We found that our toolkit approach was competitive with Maui when author-embedded keyphrases were removed from the text, a situation that we believe is consistent with our expected TLP applications. For the SemEval article collection, our toolkit approach using a Naïve Bayes classifier, Toolkit(NB), outperformed Maui as determined by *F*-measures of 20.8% versus 18.8%, respectively.

Our results with the TRC-JCT article collection when the author-provided keyphrases were present showed that the use of the first occurrence of a candidate keyphrase as a feature leads to better performance. We believe, however, that these results are misleading for TLP applications because they will tend to be biased towards discovering author-provided keyphrases in training data (such as technical articles) that contain them.

The article collection size affected the performance of both keyphrase extraction approaches. While we obtained good results using advanced decision tree-based classifiers with our toolkit approach and the TRC-JCT data set, these methods performed poorly on the SemEval data set. We found that the Naïve Bayes classifier gave the most consistent results across collection size with our toolkit approach.

Given our encouraging results relative to Maui, we plan to implement a version of our toolkit as a Python package. We will evaluate our approach against larger and more varied sets of technical documents. We will also investigate the relationship between the size of and the number of topics in a collection, and the corresponding keyphrase extraction performance. We expect this to lead to heuristics that will enable us to better curate documents in support of our technical language-based annotation efforts.

## 8 Appendix A: Obtaining the TRC Data

The data described in Sec. 3.1 contains copyrighted material that we are unable to share. They can, however, be re-created. To enable this, we prepared a data set titled *"Training and Test-Related Data for Keyphrase Extraction for Technical Language Processing"* (https://doi.org/10.18434/M32161) which is available from the NIST Public Data Repository^3^ 3 https://data.nist.gov/od/id/mds2-2161 ^4^https://trc.nist.gov/ as a compressed tar archive. This archive contains files obtained from the NIST TRC in Boulder, CO^4^.

The subdirectories keyphrase-extraction-jct-train and keyphrase-extraction-jct-test contain a total of 1153 ThermoML fles which are each associated with a corresponding Journal of Chemical Thermodynamics (JCT) article. These ThermoML fles contain information about these papers in XML format including the titles, authors, abstract, DOIs, and keyphrases. They also contain thermophysical property data unrelated to the keyphrase extraction study.

Readers wishing to replicate this work will also need to obtain the original JCT articles from the Journal of Chemical Thermodynamics.^5^ 5 https://www.sciencedirect.com/journal/the-journal-of-chemical-thermodynamics These articles can be accessed via the DOI links provided in our archive file.

## 9 Appendix B: Obtaining the SemEval-2 Data

The SemEval-2 data are available from either the SemEval-2 website^6^ 6 https://semeval2.fbk.eu/semeval2.php or from Alyona Medelyan's GitHub repository.^7^ 7 https://github.com/zelandiya/keyword-extraction-datasets We chose to use the latter because it provided the data structured in a manner suitable for use by the Maui automatic topic indexer (see Sec. 3.3).
